# Non-Linear Electrohydrodynamics in Microfluidic Devices

**DOI:** 10.3390/ijms12031633

**Published:** 2011-03-03

**Authors:** Jun Zeng

**Affiliations:** Hewlett-Packard Laboratories, Hewlett-Packard Company, 1501 Page Mill Road, Palo Alto, CA 94304, USA; E-Mail: jun.zeng@hp.com; Tel.: +1-650-857-6253

**Keywords:** dielectrophoresis, electrohydrodynamics, electrowetting, lab-on-a-chip, microfluidics, modeling, numerical simulation, reflective display

## Abstract

Since the inception of microfluidics, the electric force has been exploited as one of the leading mechanisms for driving and controlling the movement of the operating fluid and the charged suspensions. Electric force has an intrinsic advantage in miniaturized devices. Because the electrodes are placed over a small distance, from sub-millimeter to a few microns, a very high electric field is easy to obtain. The electric force can be highly localized as its strength rapidly decays away from the peak. This makes the electric force an ideal candidate for precise spatial control. The geometry and placement of the electrodes can be used to design electric fields of varying distributions, which can be readily realized by Micro-Electro-Mechanical Systems (MEMS) fabrication methods. In this paper, we examine several electrically driven liquid handling operations. The emphasis is given to non-linear electrohydrodynamic effects. We discuss the theoretical treatment and related numerical methods. Modeling and simulations are used to unveil the associated electrohydrodynamic phenomena. The modeling based investigation is interwoven with examples of microfluidic devices to illustrate the applications.

## 1. Introduction

Advances in the field of microelectronics, especially its fabrication technology, have made possible the miniaturization of fluidic components and systems, thus the discipline of microfluidics. The inkjet printhead is the earliest, and so far one of the greatest, commercial successes of microfluidics [1]. Despite the movement toward a “paperless” world for the last 30 years, over 100 million inkjet chips are produced every year to respond to the growing demand for simple, low-cost, high-quality inkjet printers. Today, inkjet printing, as one of the major digital printing enablers, is expanding into a much larger commercial and industrial printing arena.

In the early 1990s, Manz *et al.* developed the concept the of miniaturized total analysis system (μTAS) [2] based on manipulation of fluid flow at micro-length scale. Since then, the miniaturization of biochemical devices and processes, or lab-on-a-chip, has attracted great attention and has made remarkable progress. Today, enabling biochemical analysis is the dominating application in the field of microfluidics. Microfluidics is also gaining momentum in display applications (e.g., [3]).

Since the inception of microfluidics, the electric force has been exploited as one of the leading mechanisms for driving and controlling the movement of operating fluid and charged suspensions (e.g., biochemical species, beads, pigmented particles). The electric force has an intrinsic advantage in miniaturized devices. Because the electrodes are placed over a small distance, from sub-millimeter to a few microns, a very high electric field, in the order of *MV/m*, is rather easy to obtain. In addition, the electric force can be highly localized force, with its strength rapidly decaying moving away from the peak. This makes the electric force an ideal candidate for precise spatial control. The geometry and placement of the electrodes can be used to design electric fields of varying distributions, which can be readily realized by MEMS fabrication methods. Electric control also possesses advantages in system integration and reliability. For instance, there are no mechanical moving parts, and the system can be directly controlled through software.

When exposed to an external electric field, free charges will migrate due to the Coulomb force. Charges bound in molecules, both the molecules of the carrier liquid and of the biochemical species, will undergo distortion of the molecular charge density, or polarize. The volumetric force density of electric origin can be expressed [4],

(1) $${\text{f}}^{e}=\rho_{e}\text{E}-\sum_{i=1}^{m}\alpha_{i}\;\nabla (\frac{\partial W}{\partial \alpha_{i}})$$

where *E* is the electric field, *ρ*_e_ the volumetric density of the free charge, *W* the volumetric density of the electroquasistatic energy, and *α*_1_, *α*_2_, …, *α*_m_ the material properties. The first term is the Coulomb force density originating from free charges. The second is the dielectrophoresis force density originating from the bounded (paired) charges. Techniques that exploit the first forcing term are referred to as *electrophoresis*; techniques that exploit mainly the second are referred to as *dielectrophoresis*.

One critical feature of microfluidic devices compared to their macroscopic counterparts is their large surface-to-volume ratio. Forces associated with surfaces (both interfacial surfaces between solids and fluids and those between two fluids) usually are large compared to forces associated with volumes. The zeta-potential of an interfacial surface between solid substrate and electrolyte induces the formation of an electric double layer inside of which exist excessive free charges. When exposed to an electric field with a non-zero component parallel to the surface, the free charges will move and result in a significant pumping force for the electrolyte liquid. The technique exploiting the physicochemical properties of solid-electrolyte interface as described above is referred to as *electroosmosis*. Surface tension forces dominate the microfluidics when immiscible fluids are present simultaneously. When the immiscible fluids possess different electric properties, the electric field can inject electrostatic energy into the interface to cause contact angle reduction. Such force is rather strong and can result in fluid movement in the order of 25 cm/s [5]. The techniques exploiting the wetting force via electric means are referred to as electrowetting. By inserting a thin insulation layer between the fluids and the electrodes and operating with an AC drive voltage, electrolysis can be avoided. This particular configuration has been found most promising; it is referred to as *electrowetting-on-dielectric* (EWOD) [6–9], also known as *electrowetting on insulator coated electrodes* (EICE) [10].

Electrophoresis and dielectrophoresis primarily harvest the electrically generated body force; electroosmosis utilizes surface forces at the solid-electrolyte interface, and EWOD modulates wetting forces exerted at the tri-phase contact line. From applications’ perspective, electrophoresis and dielectrophoresis have been largely used to manipulate (transport, separate, or concentrate) charged or polarizable particles (e.g., biochemical species, pigmented particles). EWOD and dielectrophoresis have been applied to manipulate the operating liquid, the carrier of the biochemical species. Electrophoresis and electroosmosis are also referred to as electrokinetics [11].

In this paper, we describe several state-of-the-art approaches of electrically controlled microfluidics. In particular, we emphasize novel applications that utilize non-linear electrohydrodynamic effects. Linear electrokinetics has resulted in significant success of early generations of lab-on-a-chip, especially on-chip separation. There is a large body of literature describing the theory and application of linear electrokinetics and its application to microfluidics. Microfluidic devices using non-linear electrohydrodynamic effects are more recent and are making significant progresses in diverse areas such as life sciences (e.g., general-purpose, dynamically reconfigurable biochemical analyzer) and consumer electronics (e.g., reflective display). The literatures on non-linear electrohydrodynamic effects are growing and are scattered in different application fields. Through this paper, we strive to synthesize the seemingly different applications under a unified theoretical framework and provide a systematic quantitative treatment.

Modeling and simulations are used to unveil the associated electrohydrodynamic phenomena. We outline a unified modeling approach derived from the theoretical underpinnings of electrohydrodynamics in the next section. This approach integrates the Navier Stokes equations with the electrohydrodynamic force, Nernst-Planck equations for the transport of multiple charged suspensions, and a truncated version of Maxwell’s equations under the electroquasistatic assumption to account for the electric presence of the operating fluid and the charged suspensions. We also discuss modeling issues associated with microscopic length scale and the deformation and topological change of a two-fluid interface.

The subsequent section focuses on devices that exploit interfacial electrohydrodynamics. Examples include EWOD driven digital microfluidics, novel application of dielectrophoresis, and electro-spray ionization process that interfaces a micro total analysis system and mass spectrometry.

The section following that discusses non-linear electrokinetics. We consider the phenomenon of electro-hydrodynamic instability using the electrophoretic image display (EPID) as an example. We use modeling to illustrate the novel electrokinetics involved, the induced electrokinetic instability, and possible means to overcome it.

This paper focuses on microfluidic component technologies and the associated physicochemical phenomena. Only through enabling system level architecting and integration can microfluidics release its full application potential. While addressing the system level design issues is beyond the scope of this paper, we touch upon the systems design and integration in the conclusion section to provide a more complete picture.

## 2. Theory

The continuum assumption holds for microfluidics [12,13]. Excluding a few exceptions (e.g., piezoelectric inkjet), the compressibility of the operating liquid can be considered effectively zero. The Navier-Stokes equations can be applied to describe the hydrodynamics aspect of the microfluidics,

(2) $$\begin{array}{l} \nabla \cdot \text{u}=0\\ \rho \left(\frac{\partial \text{u}}{\partial t}+(\text{u}\cdot \nabla )\text{u}\right)=-\nabla p+\mu \nabla^{2}\;\text{u}+\text{f} \end{array}$$

where *ρ* is the fluid density, *μ* the viscosity, *u* the fluid velocity, *p* the pressure, and *f* the body force density, for instance, of electric origin (Equation (1)).

Microfluidics typically operate at a small length scale (less than 1 mm) and low frequency (≪1 GHz). Consequently, the electric field and the magnetic field decouple. The truncated version of Maxwell’s equations under the electroquasistatic assumption [14], Poisson equation, can be applied to solve the electric field,

(3) $$\nabla^{2}\;\varphi =-\frac{\rho_{e}}{ɛ}$$

where *φ* is the electric potential, ***E*** = −∇*φ*, *ɛ* the electric permittivity of the medium, and *ρ*_e_ the same as that defined in Equation (1), the volumetric density of the free charge.

The coupling between the hydrodynamics and electric field is bi-directional. The presence of the electric field adds an additional force density (Equation (1)) to the momentum equation’s right-hand side. Simultaneously, the movement of material (e.g., fluids, charged or polarizable particles) alters the electrical property distribution *ɛ* and free charge distribution *ρ*_e_—both are functions of space—hence the electric field.

For most applications concerned here, a set of charged particles may be present simultaneously. They collectively contribute to the free charge density, $\rho_{e}=F\sum_{k}z_{k}C_{k}$, where *k* is the index of the charged particles (species), *C*_k_ is the volumetric concentration, *z*_k_ the valence, and *F* the Faraday constant.

The charged particles are usually small, with diameters varying from several hundred nanometers to a few microns. For each charged particle k, its migration is governed by the Nernst-Planck transport [11],

(4) $$\begin{array}{l} \frac{\partial C_{k}}{\partial t}+\nabla \cdot {\text{j}}_{k}=0\\ {\text{j}}_{k}=-\mu_{k}z_{k}{\text{FC}}_{k}\nabla \varphi -D_{k}\nabla C_{k}+C_{k}\text{u} \end{array}$$

Here the species flux *j*_k_ accounts for the contributions from electromigration, diffusion and convection with the fluid flow. *μ*_k_ is the electro-mobility and *D*_k_ the diffusivity, *D_k_* = *μ_k_RT* according to Nernst-Einstein relation.

In the cases where immiscible fluids are present, a hydrodynamic constraint in the form of an interfacial stress boundary condition [15] is applied at the two-fluid surfaces accounting for the interfacial tension,

(5) $$({\text{T}}_{a}-{\text{T}}_{d})\cdot \text{n}+\nabla \gamma -\gamma \;\text{n}(\nabla \cdot \text{n})=0$$

where *T* is the stress tensor, n the unit normal of the interface, and *γ* the interfacial tension coefficient. The subscripts *d* and *a* denote the properties of the two immiscible fluids.

The deformation and topological change of the two-fluid interface commonly occur in microfluidic devices, such as merging of multiple droplets and splitting of a droplet. This results in an additional constraint when selecting appropriate numerical methods and tools. The Lagrangian schemes explicitly track the interfaces using interface-adaptive meshes, examples are boundary-integral and finite-element methods. This type of numerical approaches cannot simulate the topological change of the interface in a natural way. The Lagrangian-Eulerian front-tracking method is highly accurate and can handle the topological change. However, the complexity of the associated interface reconstruction algorithms is the major drawback.

From the perspective of optimal balancing among accuracy, efficiency and practicality, the front-capturing methods are the favorite of the practitioners. There are two leading front-capturing methods: volume-of-fluid and level-set. Both deliver accuracy and handle the interfacial topological change. Volume-of-fluid methods may introduce undesired spurious currents if a lower order interface reconstruction algorithm is used. The level-set method may fail to conserve mass in areas of high curvature. Today, almost all the leading commercial simulation packages implement some variations of these two methods. Examples include FLOW-3D (www.flow3d.com), CoventorWare (www.coventor.com), COMSOL (www.comsol.com), CFD-ACE+ (www.esi-group.com/products/Fluid-Dynamics/cfd-ace), and FLUENT (www.fluent.com).

The interfacial simulation solutions presented in Figures 2–4 are generated by FLOW-3D (Flow Science, Santa Fe, NM, USA) and CoventorWare (Coventor, Cambridge, MA, USA), where a volume-of-fluid method is used. In addition, Figure 6 is generated by a simulation code implemented using Matlab (MathWorks, Natick, MA, USA), and Figure 7 by ComSol (ComSol, Burlington, MA, USA).

## 3. Interfacial Electrohydrodynamics

**Figure 1.**
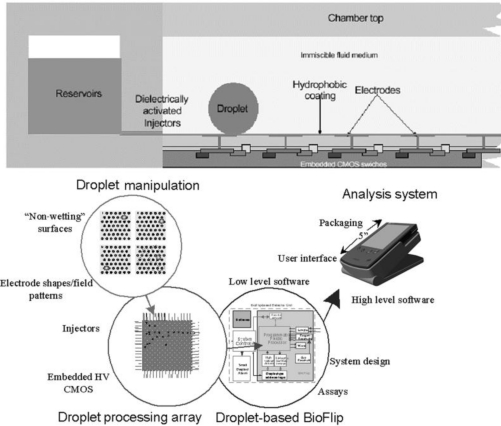
Example of droplet-based digital microfluidics architecture. Above is an elevation view showing the layered structure of the chip. Below is a diagram illustrating the system (Adapted from [4]).

The early generation of microfluidics relied on continuous fluid stream for sample transport and synchronization. This was rather different from macroscopic practices that typically use fluid boluses. In the late 1990s, droplets, pico-liter to micro-liter in size, have been identified as the microfluidic counterpart of the test tube. Droplet-based microfluidics use droplets to compartmentalize samples. Complex sample analysis procedures are built up through combining and reusing a finite set of basic droplet operations. This droplet-based microfluidics architecture is referred to as digital microfluidics. Figure 1 illustrates example digital microfluidics architecture. From operation’s perspective, digital microfluidics parallel how a typical analytical lab functions. Therefore, it is possible to map traditional bench-top protocols onto the digital microfluidics platform. Another distinctive advantage of digital microfluidics is the hierarchical architecture. A common, finite set of components and component level operations can be synthesized into numerous, complex analytical functions.

The basic operations required in digital microfluidics include metering discrete droplets [16] of unit volume from sample reservoir, transporting droplets to desired locations, merging droplets, and separating droplets into smaller ones. Droplet operations can be achieved via an array of different techniques such as thermo-capillarity and surface acoustic waves. EWOD has been developed furthest in terms of demonstrating on-chip applications that are clinically relevant [17].

In EWOD, droplets are placed onto a thin insulating layer preventing the droplets from direct contact with the electrodes. The droplets are surrounded by an immiscible liquid to prevent sample adsorption onto the hydrophobic layer and mass loss due to evaporation. The electrostatic energy stored in the thin insulating layer changes abruptly at the tri-phase contact line due to the difference of the electric properties between the droplets and the surrounding liquid. The gradient of the electrostatic energy stored in the insulating layer produces an electric force that acts on the contact line and induces contact angle reduction. The magnitude of this force, the EWOD force, can be described as [4]

(6) $$\text{f}=\frac{{ɛ}_{i}V^{2}}{8d}\left(\frac{1}{{\left(1+\frac{{ɛ}_{i}}{{ɛ}_{d}}\frac{D}{2d}\right)}^{2}}-\frac{1}{{\left(1+\frac{{ɛ}_{i}}{{ɛ}_{a}}\frac{D}{2d}\right)}^{2}}\right)$$

where *V* is the applied voltage, *D* represents droplet dimension, *d* is the thickness of the insulating layer, and *ɛ_i_*, *ɛ_d_* and *ɛ_a_* are electric permittivity of the insulating layer, the droplet and the surrounding liquid, respectively. This expression shows the force magnitude is proportional to *ɛ_i_V*^2^/*d*. The value within the (.) in Equation (6) approaches one, the maximum, when *ɛ_a_* / *ɛ_d_* ≪ 1 and *d* / *D* ≪ 1. In practice, the droplets are either conductive or highly polarizable [5] (e.g., aqueous based), and the surrounding medium is usually a non-polar solvent, that is, *ɛ_d_* ≫ *ɛ_i_* and *ɛ_a_* ≈ *ɛ_i_*; the thickness of the insulating layer is orders of magnitude smaller than the size of the droplets, *D* ≫ *d*. If we write *δ* ≡ (*ɛ_a_* / *ɛ_i_*)(*d* / *D*), Equation (6) can be re-written as

(7) $$\text{f}\approx \frac{{ɛ}_{i}V^{2}}{8d}\left(1-\frac{1}{2}\frac{{ɛ}_{a}/{ɛ}_{d}}{\delta }-4\delta^{2}+4\delta^{3}\right)$$

**Figure 2.**
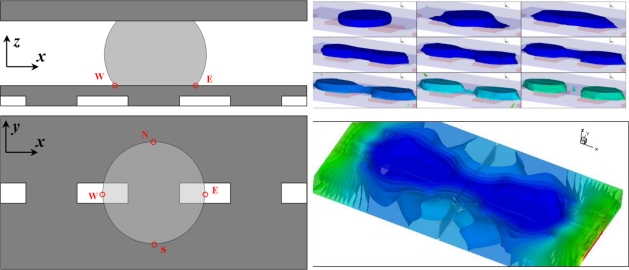
Simulation of droplet separation by EWOD. The top two figures illustrate the device configuration. Electric voltages are applied to all four electrodes embedded in the insulating material. The bottom left figure shows transient simulation solution. It illustrates the process of separating one droplet into two via EWOD. The bottom right figure shows the electric potential distribution inside the device. The color indicates the electric potential; the iso-potential surfaces are also drawn. The image shows the electric field is absent within the droplet body indicating the droplet is either conductive or highly polarizable.

Modeling methods have been applied to investigate EWOD’s device physics [4,18]. Figure 2 illustrates a simulation of separating one droplet into two [4]. Here the “pancake-shaped” droplet maintains a contact angle of 117° in the absence of a field. Upon application of 70 V to all four electrodes, the reduction of the contact angle elongates the droplet in the x direction, thereby effectively shrinking the yz-plane cross-section at the center of the droplet and eventually breaking the droplet into two parts to conclude the droplet separation. In practice, a low frequency alternating current (AC) voltage is usually applied to eliminate charge trapping [5], one of the leading failure modes of the device.

Dielectrophoresis has also been investigated as an effective operating principle for digital microfluidics [21]. Effective planar (2-D) manipulation of droplets using dielectrophoresis, similar to that using EWOD, has been demonstrated, for instance [22]. The distinctiveness of dielectrophoresis compared to EWOD is its capability to exert forces in a 3-D space. Dielectrophoretic operations do not require droplets or particles to be in contact with a solid surface (*i.e.,* reaction surface).

Most of the fluids in this discussion are incompressible and electrically linear. Therefore, the dielectrophoretic force density can be expressed as $-\frac{1}{2}{\left|\text{E}\right|}^{2}\nabla ɛ+\nabla [\frac{1}{2}(ɛ-{ɛ}_{0}){\left|\text{E}\right|}^{2}$. The first term exerts on the interface between the two immiscible fluids where there exists a discontinuity of the electric properties. The second term shows that the effectiveness of the dielectrophoresis heavily relies on the creation of a non-uniform electric field.

Dielectrophoresis’ ability to exert forces onto a free-floating medium (e.g., particle, droplet, or cell) in a 3-D space has inspired novel applications of dielectrophoresis-based microfluidics. One example is a MEMS-based micro-surgical device that can carry out axon repair for patients who suffer from serious nervous system damage [23]. MEMS-fabricated micro-knife cuts off the damaged axon regions, and then a donor axon segment is brought in to fill the gap between the ends of the host axons. Once the ends of the host and donor axons align and mate, the axon segments are fused to establish functional integrity. Dielectrophoresis is used as the operating principle responsible for bringing the donor and host axons close to each other, aligning and mating them to induce the cell fusion. In addition to being able to project forces onto free-floating axons, another dielectrophoresis-induced phenomenon critical to this application is the so-called “Pearl Chain” effect [24]. When two axons are brought to the same vicinity, a high field gradient is created between these two axon segments. Consequently, a high dielectrophoretic force is generated, pulling them closer to each other and getting them to eventually mate. This mutual attraction of axons in close quarters originates from dielectrophoresis and is of great aid in inducing axon fusion.

**Figure 3.**
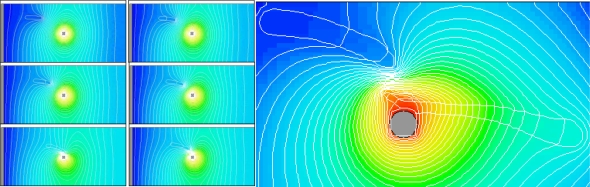
Simulation of dielectrophoresis driven axon migration. The set of small images on the left shows a transient simulation of single axon migration under an electric field generated by a pin electrode. The image on the right is a snapshot of a simulation where two axons are fused by dielectrophoresis using a pin electrode. Axons are outlined in white. Also shown are the iso-potential curves.

One of the critical design issues is the electrode shape and configuration to achieve desired electric field gradient. Figure 3 shows a simulation of dielectrophoresis driven axon migration. In this particular example, a pin electrode is used (at the center of the images) such that there is an accelerated field gradient towards the pin electrode. Iso-potential curves are overlaid on the images to show the electric field evolution. The set of images on the left of Figure 3 shows a transient simulation of axon migration. One axon, an elongated, highly polar medium, initially is placed at the upper left corner. Driven by dielectrophoresis, the axon swims towards the pin electrode following the field gradient. The iso-potential curves also show that the movement of the axon modifies (compresses) the electric field. The image on the right in Figure 3 shows one snapshot of a simulation where two axons are brought together by dielectrophoresis. The iso-potential curves are densely packed between the two axons indicating the presence of high dielectrophoresis force that will bring these two axons to meet and fuse, that is the Pearl Chain effect.

Typically, AC is used in dielectrophoresis. The AC frequency adds additional design dimension; the imaginary part of the electric permittivity exhibits frequency dependency, −*jσ*/*ϖ*, where *σ* is the conductivity and *ω* is the angular frequency of the AC field.

Under direct current (DC), the (limited) conductivity present in the liquid, in addition to the permittivity, gives rise to a phenomenon called “leaky dielectric liquid” [25,26]. Under an electric field, in addition to storing the electrostatic energy in the bulk, the liquid allows free charges to conduct through and accumulate at the interfaces between fluids. The presence of these interfacial charges results in an additional interfacial stress, especially a tangential stress, which in turn modifies the fluid dynamics.

**Figure 4.**
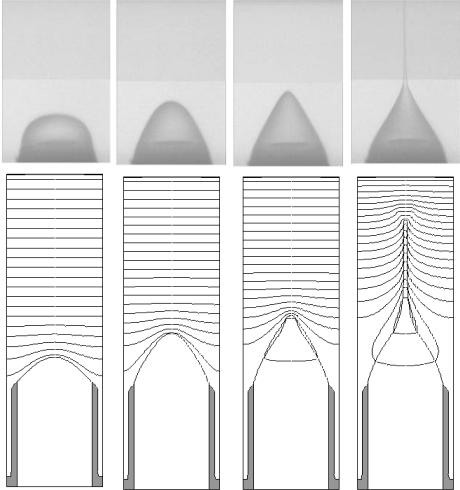
Transient sequence of the Taylor cone formation: simulation and experiment comparison. Experimental images are shown in the top row. Simulation results are shown in the bottom row. Their correspondence is indicated by the vertical alignment (Adapted from [4]).

This phenomenon has been exploited to enable a microfluidic application, called “electro-spray ionization”, that provides a bridge between microfluidics-based sample separation and mass spectrometry [27,28]. Liquid solutions from chromatographic separation chip are directed to a conductive nozzle. A high electric field is established within the vicinity of the nozzle when a voltage bias is applied between this nozzle and a far-field electrode plate. The electrohydrodynamic forces arise primarily by the presence of the interfacial charge. They work in concert with the surface tension force and a small back pressure. Together they produce a Taylor-Cone, a capillary jet with a cone-shaped base, which narrows to a fine liquid filament of nano-scale diameter. Interfacial instabilities break this filament into charged nano-droplets, which subsequently experience evaporation and Coulomb explosion, turning it into a cloud of gas-phase ions representative of the species contained in solution. The gas-phase ions flow into a mass spectrometer for further analysis. Figure 4 shows a transient sequence of the Taylor-Cone formation, including both the experimental and simulation results. Also plotted with the simulation results are the iso-potential curves to indicate the evolution of the electrical field. The interfacial charge density (surface density) *S* establishes a field discontinuity at the interface according to $S=\vec{n}\cdot ({ɛ}_{d}\nabla \varphi_{d}-{ɛ}_{a}\nabla \varphi_{a})$. The iso-potential curves surrounding the nano-jet indicate the field discontinuity at the nano-jet, thus the presence of the interfacial charge.

From a mathematical perspective, one additional equation is required to close this new, leaky-dielectric fluid boundary condition at the interface, $\text{dS}/\text{dt}=-\vec{n}\cdot \sigma \nabla \varphi_{d}$.

## 4. Non-Linear Electrokinetics

In the section above we have examined several electrohydrodynamic phenomena: in EWOD, electric field exerts its influence through the tri-phase contact line; in dielectrophoresis, the electric force shows up as both bulk force density and the surface force density on the interfaces; in electro-spray ionization, the electric force exerted itself on the interfacial surface due to the presence of the interfacial charge, the primary force for the generation of nano-jet. In addition to the simultaneous presence of media of multiple phases, there is another common theme among these phenomena and the derived applications. The electric field manipulates the movement of the operating fluid (e.g., droplets or liquid jets), and that in turn shapes the movement and presence of the species the liquids carry. For instance, the merger of droplets driven by EWOD triggers the chemical reaction between the chemical compounds encapsulated inside each droplet.

In this section we look into electrokinetic applications where the influence of the electric field is directly exerted on the species. Electrokinetics is perhaps one of the most broadly applied microfluidic operating principles. Typically voltages are applied to various ends of interconnected microfluidic channels. Aqueous-based electrolyte carrying charged species (e.g., macro-molecules, beads, or pigmented particles) fills the microfluidic channels. The electric double layer (EDL) is developed near the channel wall due to the surface charges at solid-liquid interfaces. Typically the EDL is very thin compared to the channel width. Therefore, the Helmholtz-Smoluchowski velocity is used to effectively represent its impact [11], $u_{s}=-\frac{ɛ\zeta }{\mu }E_{∥}$, where *ξ* is the zeta-potential and *E*_∥_ the electric field component parallel to the surface. For the charged species, a common practice is to assume a constant *μ*_ep_, the electrophoretic mobility, and express the particle migration velocity as ***U*** = *μ_ep_****E***. Since the electric impact on both the solid-liquid surface (electroosmosis) and the charged species (electrophoresis) is linear with respect to the electric field, this type of applications and modeling treatment is called *linear electrokinetics*.

**Figure 5.**
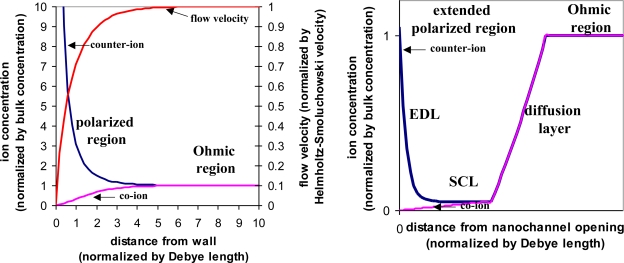
Equilibrium *vs.* non-equilibrium electroosmosis. Left: concentration polarization of the equilibrium electroosmotic flow. The normalized curves are counter-ion concentration, co-ion concentration, and the velocity of the electroosmotic flow, obtained from numerical simulation. Right: schematic representation of the concentration polarization of the non-equilibrium electroosmosis. The curves are the normalized concentrations of the counter-ions and co-ions.

In recent years, non-linearity in electrokinetics has been exploited. A nanofluidic channel, dimension of which is close to the EDL, is connected to the microfluidic channel from the side. When applying an electric field through the nanofluidic channel, which is normal to the EDL, ionic current is induced in the nanofluidic channel and effectively creates free charges within the EDL. Once this normal electric field exceeds a certain threshold, the formation of the EDL is dictated by this normal electric field instead of the intrinsic zeta-potential. Consequently, the electrokinetic effects now are characterized by the product of the normal electric field and the parallel electric field, which is no longer linear with the electric field but rather proportional to the product of two electric fields [29]. The polarized region extends from the EDL to include a space charge layer (SCL) (Figure 5). This region can block the microfluidic channel and act as an energy barrier to force charged species to accumulate. With the assistance of the parallel electric field, extremely high sample concentration can be achieved [30,31].

Non-linearity exists not only in electroosmosis as outlined above, but also in electrophoresis. In electrophoresis-based display applications, the electrophoretic ink is composed of non-polar solvents and pigments. The presence or absence of color at one pixel is achieved by the movement of pigments controlled by the applied electric field [32,33]. In non-polar solvents, the electrostatic barrier to charging is orders of magnitude higher than that in an aqueous liquid, and the ion self-energies are much greater than the thermal energies. Since charge generation is not automatic, nano-scale charge stabilizing aggregates, such as reverse micelles formed by surfactant additives, are required to initiate the charges and to stabilize the charges on the particle surfaces. This particle charging mechanism is chemical in nature, but a quantitative theory does not yet exist [34,35]. Charged pigmented particles migrate driven by the Coulomb force, overcoming the viscous drag. Simultaneously free-floating reverse micelles carrying either counter-charges or co-charges also migrate electrophoretically. In addition, the charge movement induces the liquid flow. Consequently, the governing equations include Navier-Stokes equations (Equation (2)) for solvent dynamics, Poisson equation (Equation (3)) for electric field accounting for all charges in solution, and Nernst-Planck (Equation (4)) for migration of charged species including pigmented particles, counter-charges and co-charges carried by reverse micelles. The non-linearity of electrophoresis arises from the electromigration and fluid flow convection, the first and third terms of the species flux expression in Equation (4).

**Figure 6.**
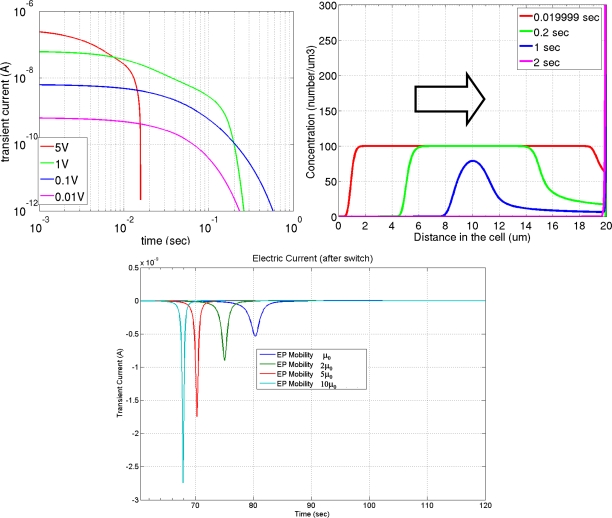
Simulation of charge screening effect using a parallel-plate cell. Top-left image shows the electric current as function of time and driving voltage, top-right image shows the evolution of the species concentration as function of time and space, the bottom image shows the electric current readout after switching the applied voltage.

The movement of counter-charges and co-charges carried by reverse micelles weakens the electric field felt by the charged particles. This charge screening effect is augmented by the possible electrochemical reactions that occur both in the bulk and on the electrode surface (in the cases where the electrodes are not insulated from the ink). Figure 6 shows a simulation of such charge screening. In this particular example, the electrophoretic ink is filled between two parallel electrode plates. Once a voltage difference is applied between the electrodes, the charged species will migrate electrophoretically and accumulate at the surfaces of the electrodes. The top left image of Figure 6 shows an electric current extracted from one electrode surface and at right the evolution of species concentration. The top-right image shows the effect of the strong charge screening [33], most applicable to this application. The electric field induced by the free-floating space charges is rather large compared to the ambient electric field, and the movement of the free-floating space charges significantly reduces the electric field felt by the pigmented particles. A concentration reduction at the transport front is observed owing to the continuously weakened electric field by charge accumulation at the electrode surface. Switching the voltage after all the charged particles are compressed against the electrodes generates a pulse of the electric current readout. The magnitude of the pulse corresponds to the mobility of the charged species. The bottom image shows the calculated current pulses using different mobility values. This electric current pulse can be measured experimentally, and is an effective way to interrogate the electric properties of the ink. The effect of charge screening poses an ink formulation optimization challenge to the surfactant additive design. Sufficient reverse micelle concentration is required to plant and stabilize particle surface charge (above the critical micellar concentration); on the other hand, low reverse micelle concentration is favored to minimize the negative impact of the charge screening.

**Figure 7.**
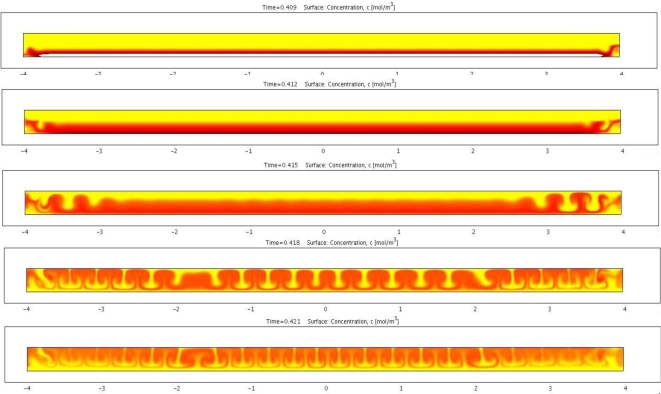
Transient simulation of electrohydrodynamic instability and the development of the cellular convective flow pattern.

The non-linearity rooted in the electromigration and the flow convection gives rise to the electrohydrodynamic instability. Figure 7 shows a transient simulation of the electrophoretic migration of charged species including charged pigmented particles and reverse micelles. The color indicates the concentration of the pigmented particles. Simulation shows the initially planer front gradually developing into a cellular convective flow pattern. This type of electrophoretic migration pattern has been observed repeatedly in experiments. The experimental images show qualitative agreement with the simulation results. This type of flow in general is not beneficial to the particle mobility since the flow direction is not always aligned with that of the particle. An analysis based on the perturbation method was performed to identify the condition that triggers such instability. This analysis showed that for an applied voltage above a certain threshold, such instability is likely to occur. Higher particle surface charge density and/or larger particles can raise this threshold. Furthermore, certain geometrical design of the cell can help to stabilize the electrophoretic migration.

## 5. Conclusions: Enabling the Systems

Since the inception of microfluidics, the electric force has been exploited as an important mechanism for driving and controlling the movement of the operating fluid and charged suspensions. In this paper we examined several electrically driven liquid handling operations and discussed the theoretical treatment and numerical methods. Even though many examples were drawn from the life sciences, the methods described are much more widely applicable. For instance, EWOD has found applications in imaging and printing [36].

This paper focuses on detailed physical simulations of component-level operations, which are the key enablers. However, to fulfill certain desired services, such as agent detection with portability, durability and reliability requirement, the components must be synthesized to form a functional system. Consequently, system-level complexities need to be addressed. This includes architectural level designs that decompose the application into a finite set of operations, mapping the operations to hardware components, and component placement and routing (both electrically and fluidically). It also includes executing level designs that determine runtime workflow management, built-in self testing, fault detection and tolerance [37,38]. It also includes a chip-to-world interface.

From a computer-aided systems design perspective, enabling the system design calls for higher level of abstraction above the detailed physical simulations presented here. Such synthesis uses operational models, or behavioral models, to encapsulate the component-level complexities, provide common component interfaces over diverse operating principles, and enable hierarchical modeling of a system composed of heterogeneous, concurrent components. Enabling system level modeling is the key area of focus for the microfluidics modeling community. Compared to the state of the art of computer-aided design for microelectronics, the modeling aid for microfluidics system design and integration is far less mature and presents a significant challenge and thus opportunity.
